# Freins et réticences à la vaccination pédiatrique (PEV) et contre la Covid-19 : résultats d'une enquête au Niger

**DOI:** 10.48327/mtsi.v4i3.2024.431

**Published:** 2024-07-03

**Authors:** Bernard SEYTRE, Sanoussi CHAIBOU, Emmanuel CHABOT, Bernard SIMON

**Affiliations:** 1 bnscommunication, 37 rue Gnati, Klikamé, Lomé, Togo; 2 Université Laval, Québec, Canada; 3 Croix-Rouge française, 98 rue Didot, 75014 Paris, France

**Keywords:** Vaccination, PEV, Covid-19, Représentations, Hésitation vaccinale, Niger, Niamey, Gaffati, Tirmini, Afrique subsaharienne, Vaccination, EPI, Covid-19, Perceptions, Vaccine refusal, Niger, Niamey, Gaffati, Tirmini, Sub-Saharan Africa

## Abstract

**Introduction:**

Nous avons mené au Niger une enquête socio-anthropologique alliant les méthodes quantitative et qualitative de collecte de données sur les représentations de la population sur la vaccination, afin de fournir les bases à une stratégie de communication pour promouvoir les vaccins du Programme élargi de vaccination (PEV) et contre la Covid-19.

**Matériel et méthode:**

Pour l’étude quantitative, nous avons interviewé 509 personnes, choisies selon la méthode des quotas, à 80 % dans la capitale pour des raisons sécuritaires et logistiques. Le volet qualitatif a porté sur 10 personnes en entretiens individuels approfondis et 30 en groupes focus (GF).

**Résultats:**

Nos résultats montrent une adhésion quasi unanime à la vaccination pédiatrique. La crainte des effets secondaires, mentionnés par 30,6 % des enquêtés, un manque d'amabilité du personnel de santé et le temps d'attente, mentionnés respectivement par 21,4 % et 16,9 %, apparaissent comme des facteurs contribuant à expliquer un abandon après les premières doses et le non-respect du calendrier vaccinal. La faible adhésion à la vaccination contre la Covid-19 s'explique par la conjonction de plusieurs idées erronées sur la pandémie. D'abord, la majorité des enquêtés pense que la maladie n'est pas présente dans le pays, 43,8 % seulement estimant qu'elle l'est. Ensuite, la quasi-totalité croit que seules des personnes malades transmettent le virus responsable de la Covid-19; 12,8 % estiment connaître quelqu'un qui a eu la maladie et 5,1 % en connaissent dans leur entourage. L'ensemble de ces représentations se traduit par une perception très faible du risque de contracter la maladie. Les rumeurs négatives qui circulent sur les vaccins pédiatriques et contre la Covid-19 ne semblent pas jouer de rôle significatif.

**Conclusion:**

Les efforts de communication sur les vaccins du PEV doivent porter sur l'explication des effets secondaires et leur gestion, ainsi que sur l'amélioration de l'accueil des parents. Concernant la vaccination contre la Covid-19, la communication doit se concentrer sur la réalité de la maladie dans le pays et les groupes à risque de formes graves.

## Abstract (en)

**Background:**

Vaccination adherence among populations is a complex process involving, on the one hand, the expected benefit of a vaccine, and on the other, the perceived risk. To develop an evidence-based communication strategy aimed at improving vaccination coverage in Niger, we conducted a mixed socio-anthropological study of the perceptions among the population on the benefit and the risk of the childhood (Expanded Program on Immunization, EPI) and the Covid-19 vaccines. While the vaccine coverage for some of the EPI vaccines is above 80%, only 33% of the children are fully vaccinated, according to the national vaccination schedule. The first objective of our study was to understand the perceptions that might explain this drop off.

The second objective was to identify the drivers of the low adherence to the Covid-19 vaccination, 22% of the illegible population being vaccinated.

**Materials and methods:**

In March 2023, we interviewed 509 people for the quantitative study and 40 people through focus groups and individual interviews for the qualitative one, in Niamey (80% of the interviewees) and two villages.

**Results:**

96.9% of the interviewees think that childhood vaccines are “a good thing,” even though 30.6% know that they might have negative side effects. While 87.8% think that it is “easy” or “very easy” to get children vaccinated, 21.4% point out as “annoying” the lack of amiability by the health care workers and 16.9% the waiting time. The qualitative study showed that these two complaints drive some women not to complete the vaccination schedule. We might also hypothesize that, given the perceived lack of amiability, some women don't get enough information on the side effects and their management. Surprisingly, 73.3% of the interviewees think that vaccines against the disease are a good thing, and 83% of those who have heard messages promoting the vaccination approve them. This apparent contradiction with the low vaccine uptake is explained by a very low perception of the Covid-19 risks. More than half of the population surveyed believe that the disease is not present in the country, a very large majority believe that only ill people can transmit the disease, while only 12.8% think they know anybody who has ever been sick with Covid-19. According to our results, the circulating rumors on the vaccines don't play a significant role in the low adherence to Covid-19 uptake, nor in the insufficient completeness of the EPI vaccination schedule.

**Conclusion:**

The communication efforts on EPI vaccination should focus on the explanation of the side effects and their management, as well as improving the organization of the vaccination sessions. The communication on Covid-19 vaccination should focus on the reality of the disease in the country and the groups at risk for severe forms.

## Introduction

Dans le cadre du programme AFROVAX de renforcement de la vaccination mis en œuvre par la Croix-Rouge française au Niger, aux Comores et en Guinée, nous avons mené au Niger une enquête socio-anthropologique quantitative et qualitative sur les représentations de la population, dont les résultats ont fourni les bases d'une stratégie et d'outils de communication visant à accroître l'adhésion à la vaccination.

AFROVAX concerne la vaccination systématique avec les vaccins du Programme élargi de vaccination (PEV) et celle contre la Covid-19, qui visent des publics différents. Les vaccins du PEV sont administrés pendant les deux premières années de la vie et tout visiteur d'un centre de santé constate que ce sont quasiment exclusivement les mères qui amènent les bébés pour cette vaccination, tandis que les vaccins contre la Covid-19 s'adressent aux adultes de plus de 18 ans. Les couvertures vaccinales des vaccins du PEV et de ceux contre la Covid-19 sont, en outre, très différentes.

En 2021, 33 % des enfants étaient considérés comme complètement vaccinés au Niger contre neuf maladies, après avoir reçu quatre vaccins et leurs rappels éventuels, au cours de six séances de vaccination [7]. Cependant, la couverture de certains de ces vaccins est considérablement plus élevée avec, par exemple, 81 % pour la première dose du vaccin pentavalent (diphtérie, coqueluche, tétanos, hépatite B et *Haemophilus influenzae* type b), 83 % pour le vaccin contre le pneumocoque et 80 % pour celui contre le rotavirus. Diverses causes peuvent expliquer la différence de couverture entre certains vaccins et la vaccination complète, dont les ruptures de stocks de vaccins ou d'autres perturbations de l'offre et, au niveau de la demande, le manque de respect du calendrier vaccinal par les mères. Le taux d'abandon spécifique entre la première et la troisième dose des vaccins qui nécessitent des rappels est évalué à 36 % pour le vaccin contre la poliomyélite, 21 % pour le pentavalent et 11 % pour le vaccin contre le rotavirus. Une étude menée récemment au Bénin montre également une chute de la couverture vaccinale « à mesure que le nombre de doses augmentait », avec un maximum de 100 % pour le BCG administré à la naissance et un minimum de 12,61 % pour la troisième dose du vaccin contre le rotavirus [5].

La couverture vaccinale contre la Covid-19 est faible au Niger, comme dans la majorité des pays d'Afrique subsaharienne. Fin 2022, elle atteignait 22 % de la population cible, les adultes de plus de 18 ans [12]. Une fraction difficile à évaluer de ce taux correspond à des personnes obligées, jusqu'au 26 juin 2023, de se faire vacciner pour franchir les frontières, dans une région dont la mobilité est un trait caractéristique [2].

Envisagées du point de vue de la communication, ces données amènent aux hypothèses suivantes que notre étude cherchait à vérifier :

- vaccins du PEV : forte adhésion à la vaccination, suggérée par les taux de couverture vaccinale supérieurs à 80 %; sous-estimation de l'importance du respect du calendrier vaccinal, indiquée par les abandons auxquels pourraient contribuer la crainte des effets secondaires des vaccins ou les conditions de vaccination;
- vaccin contre la Covid-19 : la faible adhésion à la vaccination pourrait s'expliquer par une sous-estimation du risque représenté par la maladie ou une opinion négative sur les vaccins, éventuellement suscitée par des rumeurs.

## Matériels et méthodes

La collecte des données quantitatives et qualitatives a eu lieu du 10 au 27 mars 2023 dans les cinq arrondissements de Niamey, capitale et plus grande ville du Niger, et dans deux communes rurales de la région de Zinder, Gaffati et Tirmini, après deux journées de formation à Niamey de 10 enquêteurs, 4 femmes et 6 hommes, titulaires de diplômes allant de la licence au DEA. Le protocole a été approuvé par la Direction des immunisations du ministère de la Santé publique, de la population et des affaires sociales du Niger, ainsi que par des représentants de l'OMS et de l'Unicef travaillant sur la vaccination dans le pays.

Les sites ont été choisis, d'une part, car ce sont des lieux d'intervention de la Croix-Rouge française (ce qui facilitait la logistique à Zinder) et, surtout, en raison de leur accessibilité et des enjeux de sécurité, la ville de Niamey et la région de Zinder étant les zones les plus sécurisées du pays. Pour optimiser la collecte des données dans le délai imparti, les communes de Tirmini et Gaffati, proches de la ville de Zinder, ont été sélectionnées de manière raisonnée : premièrement, pour leur accessibilité géographique par la route et, deuxièmement, pour éviter de choisir des communes frontalières avec les régions de Maradi, Diffa et d'Agadez, qui sont très éloignées et où sévissent des conflits armés, avec des incursions de Boko Haram, des enlèvements et des demandes de rançon.

Vu les caractéristiques de ces enquêtes et la sensibilité recherchée, l’échantillonnage par quotas était la méthode la mieux adaptée pour l'enquête quantitative. La taille de l’échantillon a été calculée par la formule de Cochran [t^2^xpx(1-p)/m^2^] avec un niveau de confiance de 95 % (t=1,96, p=40 %, m=5 %), ce qui donne une taille de 369 personnes, que nous avons portée à 500 pour augmenter la puissance de l’étude. Sur le terrain, 509 personnes ont été interviewées. La population étudiée a été répartie en quotas par sexe et âge en fonction de la démographie urbaine et rurale : 18-24 ans, 25-59 ans, 60 ans et plus [8].

Les leaders communautaires (chefs de quartier ou de village) ont diffusé l'information sur cette étude auprès de la population. Les enquêteurs sont passés au porte-à-porte, en semaine, de 9 heures à 18 heures, pour recruter des personnes acceptant d’être interrogées et répondant aux critères, jusqu’à atteindre le nombre total prévu. Le respect des quotas était vérifié à mesure de la progression de l'enquête. Les réponses étaient anonymes. Les répartitions par localités, sexes, âges, occupations et niveaux d'instruction des 509 personnes interviewées sont indiquées dans le Tableau I.

**Tableau I:** Caractéristiques des personnes enquêtées pour l'enquête quantitative Characteristics of people interviewed for the quantitative survey

	Gaffati N=54	Tirmini N=55	Niamey N=400	Total N= 509
**Sexe**				
femmes	36 (67 %)	30 (55 %)	220 (55 %)	286 (56,2 %)
hommes	18 (33 %)	25 (45 %)	180 (45 %)	223 (43,8 %)
**Âge**				
18-24 ans	14 (26 %)	13 (24 %)	104 (26 %)	131 (25,7 %)
25-59 ans	32 (59 %)	30 (54 %)	264 (66 %)	326 (64 %)
60 ans et +	8 (15 %)	12 (22 %)	32 (8 %)	52 (10,2 %)
**Niveau enseignement**				
aucun	23 (43 %)	23 (42 %)	129 (32,3 %)	175 (34,4 %)
primaire	11 (20 %)	12 (22 %)	81 (20,3 %)	104 (20,4 %)
collège	18 (33 %)	16 (29 %)	75 (18,8 %)	109 (21,4 %)
lycée	1 (2 %)	4 (7 %)	45 (11,3 %)	50 (9,8 %)
supérieur	1 (2%)	0 (0 %)	70 (17,5 %)	71 (13,9 %)
**Activité professionnelle**				
chômeur	0 (0 %)	0 (0 %)	72 (18,0 %)	72 (14,1 %)
étudiant	1 (2 %)	2 (4 %)	52 (13,0 %)	55 (10,8 %)
retraité	0 (0 %)	1 (2 %)	10 (2,5 %)	11 (2,2 %)
sans activité	16 (30 %)	20 (36 %)	74 (18,5 %)	110 (21,6 %)
secteur informel	33 (61 %)	31 (56 %)	133 (33,3 %)	197 (38,7 %)
secteur privé	3 (6 %)	0 (0 %)	34 (8,5 %)	37 (7,3 %)
secteur public	1 (2 %)	1 (2 %)	25 (6,3 %)	27 (5,3 %)

L'ordre des questions a été déterminé pour éviter que la crainte des effets secondaires des vaccins n'induise des réponses négatives aux questions sur leur efficacité et qu'un rejet éventuel des vaccins contre la Covid-19 ne suscite des réponses négatives sur les vaccins en général. Les thèmes ont donc été abordés dans l'ordre suivant : causes des maladies infectieuses, efficacité des vaccins, connaissance des vaccins du PEV, effets secondaires des vaccins du PEV, Covid-19, vaccins contre la Covid-19. Les enquêteurs, tous diplômés en sociologie, avaient pour consigne de ne pas mentionner la Covid-19 avant la première question concernant l’épidémie. L'administration des questionnaires et la conduite des interviews ont été faites en haoussa à Gaffati et Tirmini, et en djerma et haoussa à Niamey. Les entrevues ont été retranscrites en français, puis analysées avec le logiciel de traitement des données qualitatives Dedoose.

Les réponses ont été saisies sur tablette, téléchargées sur la plate-forme ODK puis exportées sous forme de fichier Excel.

Les leaders communautaires ont facilité le recrutement des participants aux groupes focus (GF) et aux entretiens individuels de l'enquête qualitative. Une socio-anthropologue titulaire d'une maîtrise et un sociologue titulaire d'un DEA ont réuni deux GF avec des hommes et deux avec des femmes, et mené 10 entretiens individuels approfondis (Tableau II).

**Tableau II:** Effectifs de personnes interviewées et de participants aux groupes de discussion Table II: Number of individual respondents and those included in focus groups

Localités	Entretiens individuels approfondis	Groupes focus	Nombre total de personnes
Gaffati	2	1	11
Tirmini	2	1	11
Niamey	6	2	18
**Total**	**10**	**4**	**40**

Les groupes focus et entretiens ont été enregistrés, puis transcrits. Les participants ont été sélectionnés de manière raisonnée, en tenant compte de leur niveau de connaissance des sujets importants. L’échantillonnage raisonné est basé sur l'objectif de rechercher une compréhension approfondie des différentes thématiques liées à la vaccination PEV et à la Covid-19, en incluant des hommes, des femmes et des personnes âgées de plus de 60 ans, en fonction de leur âge et de leur lieu de résidence (Niamey, Tirmini et Gaffati). De plus, un accent particulier a été mis sur la diversification des catégories de répondants. Par exemple, des guides d'entretiens ont été élaborés en tenant compte de leur tranche d’âge.

## Résultats

L'enquête quantitative a porté sur 509 sujets (Tableau I).

### Connaissances générales

La connaissance de l'existence des microbes et de leur rôle causal dans certaines maladies est extrêmement élevée : 90,6 % ont répondu « Oui » à la question « Savez-vous ce qu'est un virus ou un microbe? ». A la question « Que peut provoquer un microbe ou un virus lorsqu'il infecte une personne? », 98,7 % ont répondu « Une maladie » (les autres réponses possibles étaient « Une bonne chose », « Rien », « Ne se prononce pas »).

### Vaccins du PEV

Il a été répondu « Oui » par 96,9 % des participants à la question « Est-ce une bonne chose de faire vacciner un jeune enfant? ». A la question « Quel est le but de la vaccination des jeunes enfants? », qui proposait plusieurs réponses possibles, 97,1 % ont choisi qu'elle protège contre les maladies (Tableau III).

**Tableau III:** Réponses concernant le but de la vaccination des jeunes enfants Responses concerning the purpose of vaccinating young children

Protéger des maladies	97,1 %
Prendre du poids	7,7 %
Aider à grandir	20,8 %
Ne sait pas	2,8 %

Le vaccin oral contre la poliomyélite ayant suscité des résistances dans le nord du Nigéria [14], frontalier avec le Niger, nous avons demandé si le vaccin oral contre la poliomyélite était une bonne chose : 88,4 % ont répondu « Oui » et 9,8 %, « Non ».

Sur 509 interviewés, 156 personnes (soit 30,6 %) ont répondu « Oui » à la question « Pensez-vous que certains vaccins des jeunes enfants ont des effets négatifs pour l'enfant? ». A ces 156 personnes, nous avons demandé « Quels types de vaccins ont des effets négatifs pour l'enfant? », avec plusieurs réponses possibles : 73,7 % ont répondu « Les vaccins injectables » et 37,8 % « Le vaccin oral contre la poliomyélite ».

Dans les groupes focus de Niamey et de Zinder, des femmes ont mentionné leur inquiétude à propos des effets secondaires des vaccins, qui amènent certains parents à ne pas faire vacciner leurs enfants. Il ressort des propos tenus un manque d'information de la part du personnel de santé.

« *Malgré l'importance de ces vaccins préventifs, certains parents cachent leurs enfants en cas de campagne de vaccination car ils en ont décidé ainsi ou parfois à cause des rumeurs qui disent que les vaccins préventifs ont des effets secondaires et conduisent même à la mort. Tout simplement parce qu'un enfant vacciné tombe malade, ou parfois ça donne une petite plaie par coïncidence après le vaccin l'enfant meurt.* » (Entretien individuel avec une femme à Tirmini, région de Zinder)

**Figure 1:**
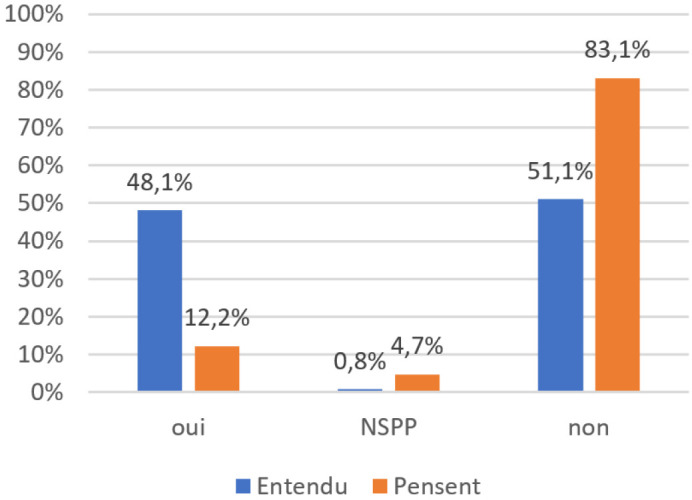
Réponses à la question « Avez-vous entendu dire/pensez-vous que des vaccins stérilisent? » (Pourcentages calculés sur l'ensemble du panel. NSPP : ne se prononce pas) Responses to the question “Have you heard/ do you believe that vaccines sterilize?” (Percentages are calculated for the entire panel. NSPP: don't know)

**Figure 2:**
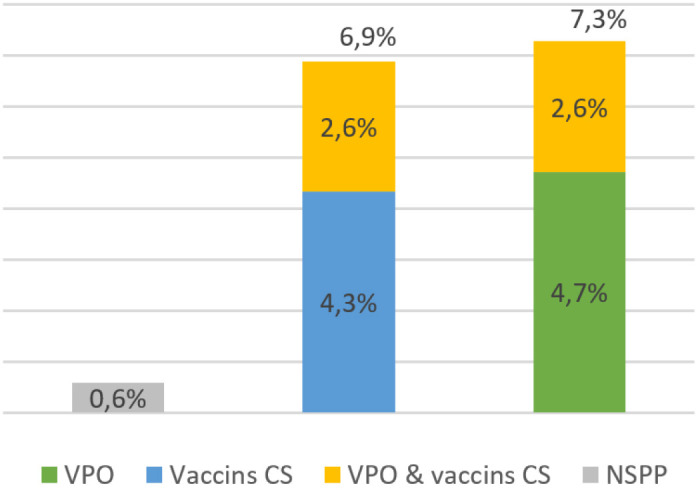
Réponse à la question « Quels vaccins rendent les filles stériles? » (Pourcentages calculés sur l'ensemble du panel. VPO : vaccin polio oral. Vaccins CS : vaccins administrés au centre de santé. NSPP : ne se prononce pas) Responses to the question “Which vaccines make girls infertile?” (Percentages are calculated for the entire panel. VPO: oral polio vaccine. CS vaccines: vaccines administered at the health center. NSPP: don't know)

« *À cause de ces effets indésirables, certains parents refusent de faire vacciner leurs enfants, en plus il y a aussi l'ignorance. Ces parents disent que les vaccins rendent les enfants handicapés et surtout pour le vaccin oral contre la poliomyélite. Les enfants qui ont reçu ce vaccin, on les traite de Diyan polio c'est à dire les enfants de la poliomyélite. Ces enfants ont la tête dure, donc très impolis.* » (Groupe focus avec des femmes à Tirmini, région de Zinder) Une rumeur circulant en Afrique selon laquelle le vaccin oral contre la poliomyélite stériliserait les enfants et des articles de presse nigériens mentionnant la même rumeur pour d'autres vaccins, nous avons cherché à mesurer son impact en posant une double question. Parmi les interviewés, 48,1 % ont répondu « Oui » à une première question « Avez-vous entendu dire que certains vaccins rendent les filles stériles? ». Puis nous avons demandé « Pensez-vous que certains vaccins rendent les filles stériles? », question à laquelle 12,2 % (62 personnes) ont répondu « Oui » (Fig. 1). Aux 62 personnes qui pensaient que des vaccins stérilisent, nous avons demandé quels vaccins, avec plusieurs réponses possibles : parmi ces personnes, 35 ont répondu « Les vaccins injectés au centre de santé » et 37 « Le vaccin oral contre la poliomyélite ». L'enquête qualitative indiquant que les parents font une distinction entre les vaccins du PEV, administrés dans les centres de santé, et le vaccin oral contre la poliomyélite, administré lors de journées de vaccination au porte-àporte même s'il est également donné avec les vaccins du PEV, la rumeur doit être analysée séparément : les vaccins du PEV seraient responsables de stérilisation pour 6,9 % de la population interviewée, le vaccin oral contre la poliomyélite pour 7,3 % (Fig. 2).

**Figure 3:**
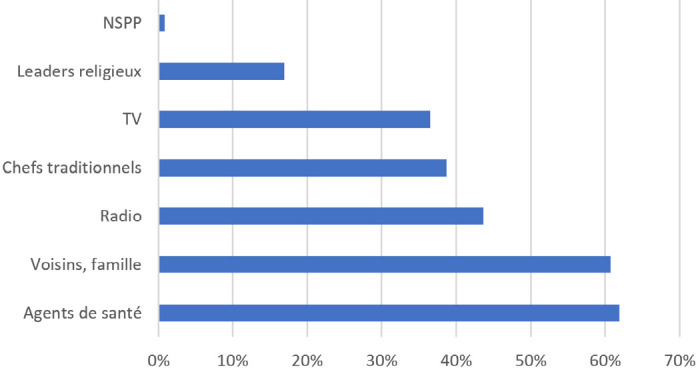
Réponses à la question « Comment êtesvous informé sur les vaccins? » (Plusieurs réponses possibles. Pourcentages calculés sur l'ensemble du panel. NSPP : ne se prononce pas) Responses to the question “How did you learn about vaccines?” (Multiple responses possible. Percentages are calculated for the entire panel. NSPP: don't know)

Nous avons questionné les interviewés sur leurs sources d'information concernant les vaccins et leur confiance dans ces sources. La Covid-19 n'ayant pas encore été mentionnée par les enquêteurs, nous pouvons avancer que les réponses s'appliquent à la vaccination pédiatrique, évoquée par les questions précédentes. Si les deux premières sources d'information sont les agents de santé (61,9 %) et les voisins/la famille (60,7 %), les premiers sont très largement considérés comme les plus fiables, avec 81,7 % de « très confiance », tandis que l'entourage est le moins fiable, avec 24,8 % de « très confiance » et 40,9 % de « très » et « un peu méfiants » (Fig. 3 et 4). Les informations recueillies auprès des voisins et de la famille sont donc moins susceptibles d'influencer les connaissances, croyances et comportements que celles transmises par les agents de santé, si nous excluons la pression sociale.

**Figure 4:**
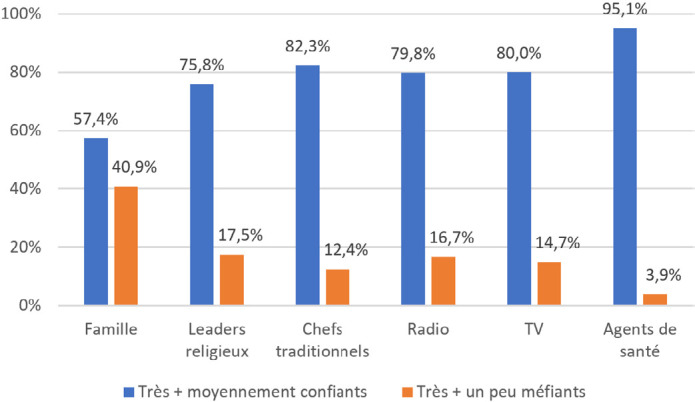
Réponses à la question « Quelle confiance avez-vous dans ce qu'ils vous disent des vaccins? » (Pourcentages calculés sur l'ensemble du panel) Responses to the question, “How much do you trust what you are told about vaccines?” (Percentages are calculated for the entire panel)

Une forte majorité de la population (87,8 %) estime que faire vacciner ses enfants est « Facile » ou « Très facile » (Tableau IV).

**Tableau IV:** Distribution du niveau de difficulté pour faire vacciner les jeunes enfants Distribution of the level of difficulty in vaccinating young children

Très difficile	0,2 %
Difficile	3,1 %
Ni facile, ni difficile	8,3 %
Facile	34,6 %
Très facile	53,2 %
Ne se prononce pas	0,6 %

Nous avons ensuite demandé quels facteurs étaient considérés comme un obstacle, avec plusieurs réponses possibles : 69,2 % estiment ne ressentir aucune gêne, les gênes exprimées étant substantiellement différentes selon les localités (Tableau V). Les 36 personnes qui ont mentionné l’éloignement résident à Niamey. Le manque d'amabilité du personnel de santé (21,4 %) et le temps d'attente (16,9 %) sont deux motifs de gêne pour faire vacciner ses enfants avec des niveaux très différents selon les lieux. Le maximum est de 31,5 % pour ces deux motifs à Gaffati et le minimum, 14 % pour le temps d'attente à Niamey.

**Tableau V:** Réponses à la question « Qu'est-ce qui est le plus gênant pour faire vacciner vos jeunes enfants? » (*Pourcentages calculés sur le nombre d'enquêtés de la localité) Responses to the question “What is the most inconvenient thing about vaccinating your young children?” ‘*Percentages are calculated based on the number of respondents in the locality)

	Gaffati*	Niamey*	Tirmini*	Total
**Rien ne me gêne, c'est facile**	36	279	37	352
	67 %	69,8 %	67 %	69,2 %
**L’éloignement du lieu de vaccination**	0	36	0	36
	0 %	9 %	0 %	7,1 %
**Le manque d'amabilité du personnel de santé**	17	82	10	109
	31 %	20,5 %	18 %	21,4 %
**Le temps d'attente**	17	56	13	86
	31 %	14 %	24 %	16,9 %
**Nombre d'enquêtés**	54	400	55	509

Le temps d'attente est un obstacle mis en évidence par l'enquête qualitative, avec des propos significatifs tels que : « *Le seul problème reste la longue attente au niveau des centres de santé avant d’être servis. Du coup certaines femmes se fâchent et quittent le lieu où se font les vaccins. Parmi elles, certaines retournent après mais d'autres ne retourneront jamais ».* (GF femmes, Niamey)

### Covid-19

**Figure 5:**
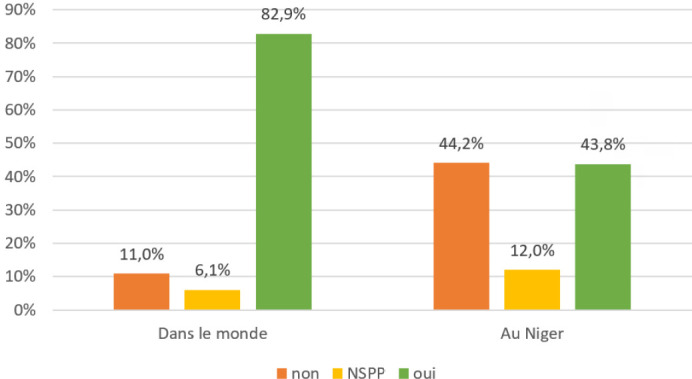
Réponse à la question « Est-ce que la Covid-19 existe? » (Pourcentages calculés sur l'ensemble du panel. NSPP : ne se prononce pas) Responses to the question “Does the Covid-19 exist?” (Percentages are calculated for the entire panel; NSPP: don't know)

Alors que la très large majorité des interviewés pense que la Covid-19 existe (82,9 %), ils sont moins de la moitié à estimer qu'elle est présente au Niger (43,8 %) (Fig. 5).

La question suivante a apporté un élément d'explication aux réponses précédentes. Des images de la maladie « dans le monde » ont été vues à la télévision par 72,5 % des enquêtés et « au Niger », par 27,1 % (71,5 % ont dit ne pas avoir vu d'images au Niger). En outre, très peu d'interviewés ont déclaré connaître directement quelqu'un qui a eu la Covid-19 (12,8 %) et encore moins dans leur famille (5,1 %). Quasiment tous les enquêtés ont « entendu parler du coronavirus » (98,2 %) et 74,3 % savent qu'un virus est la cause de la Covid-19, mais à la question « Si je vous dis que la maladie est provoquée par un virus, un coronavirus, chez qui peut-on trouver ce virus? » 3,9 % seulement ont répondu « Tout le monde » et 87,6 % « Uniquement des personnes malades ». Parmi les interviewés, 73,3 % ont répondu « Oui » à la question « Pensez-vous que les vaccins contre la Covid-19 sont une bonne chose? », 21,2 % choisissant « Non ». A ces derniers, nous avons demandé les raisons de cette opinion, plusieurs réponses étant possibles. Les deux réponses les plus choisies, « Inutiles ici car la Covid-19 n'est pas au Niger » et « Provoquent des maladies ou des décès », représentent 9,8 % et 9,6 % des 509 personnes du panel. Les autres réponses possibles ont été choisies par environ 3 % : « Aucune efficacité contre cette maladie », « Peuvent transmettre la Covid-19 », « Inutiles dans le monde entier car la Covid-19 n'existe pas ».

Nous avons ensuite posé une double question : « Avez-vous vu ou entendu des messages recommandant la vaccination contre la Covid-19? », puis à ceux qui répondaient « Oui » « Approuvez-vous ces messages? ». Les messages ont été entendus par 82,9 % et parmi ceux-ci 83,4 % les approuvaient, soit 69,2 % de l'ensemble du panel.

Nous avons posé une double question sur quatre rumeurs que nous avions identifiées comme circulant en Afrique de l'Ouest : « Avez-vous entendu *[indication de la rumeur]*? », puis « Pensez-vous que *[indication de la rumeur*]? ».

La rumeur la plus entendue et la plus admise est celle selon laquelle « les pays riches envoient en Afrique des vaccins contre la Covid-19 qu'ils ne veulent pas utiliser chez eux » : 42,4 % l'ont entendue et 26,1 % la considèrent comme exacte. Elle est suivie par la rumeur selon laquelle les vaccins « ne servent à rien et sont proposés pour permettre à l'industrie pharmaceutique de gagner de l'argent », avec 36,1 % et 21,4 %, respectivement. Les rumeurs selon lesquelles les vaccins « stérilisent les femmes » ou « injectent une puce électronique » se situent à des niveaux nettement inférieurs avec, respectivement, 23,6 % (entendue) et 7,9 % (crue) pour la première, 21,6 % (entendue) et 7,5 % (crue) pour la seconde.

## Discussion

Une limitation de la représentativité de nos résultats est le fait que 80 % des personnes interviewées pour l'enquête quantitative l'ont été dans la capitale, alors que la population du Niger est en majorité rurale. Notre échantillon comprend 56,2 % de femmes, ce qui constitue une sur-représentation par rapport aux 50,9 % de femmes dans la population adulte du Niger, mais d'après nos calculs, cela ne modifie pas significativement les résultats. Enfin, l’échantillon recruté pour l'enquête quantitative l'a été au porte-à-porte, jusqu’à atteinte des quotas par âges et sexes, ce qui est une limitation par rapport à un recrutement aléatoire.

Nos résultats montrent une certaine compréhension des maladies infectieuses, la quasitotalité des personnes interrogées disant savoir ce qu'est un virus ou un microbe (90,6 %) et qu'ils provoquent des maladies (98,7 %). C'est un point d'appui pour la sensibilisation à la prévention des maladies infectieuses, que ce soit par la vaccination, l'hygiène ou des mesures telles que l'usage d'une moustiquaire ou de masques. La quasi-totalité pense également que la vaccination pédiatrique est une bonne chose (96,9 %) car elle protège contre des maladies (97,1 %).

Deux ensembles de résultats apportent des éléments d'explication au manque de respect du calendrier vaccinal, qui se traduit par un abandon entre les premières doses et les rappels. Les premiers concernent les effets secondaires des vaccins, mentionnés par 30,6 % de la population, ainsi que le manque d'explications du personnel de santé pour avertir les mères de la manifestation possible de ces effets et leur indiquer la conduite à tenir. Les seconds portent sur l'accueil dans les centres de santé. L'accès à la vaccination pédiatrique est considéré comme facile par 87,8 % de la population, 69,2 % estimant que « rien ne la gêne », des réponses qui auraient peut-être été différentes dans des groupes nomades, non inclus dans notre étude. Cependant, 21,4 % mentionnent le manque d'amabilité du personnel de santé et 16,9 % le temps d'attente, avec des maximums de 31,5 % dans un des villages de l'enquête, deux motifs de mécontentement de la population fréquemment mentionnés par des études menées en Afrique subsaharienne [6, 9, 13]. Au Bénin, l'accueil est perçu comme mauvais ou très mauvais par 17 % des mères et le temps d'attente comme long ou très long par 65,2 % [5].

Une autre cause d'abandon pourrait être une mauvaise compréhension de la nécessité de respecter le calendrier vaccinal pour une bonne immunisation. Les parents pourraient ne pas comprendre, d'une part, que des rappels sont nécessaires pour certains vaccins et, d'autre part, que les vaccins administrés ne protègent pas contre les mêmes maladies, ce qui conduirait des femmes à considérer les dernières vaccinations du calendrier comme moins utiles que les premières. Notre étude n'apporte pas de réponse sur cet aspect qui, s'il était vérifié, renverrait à l'insuffisance des explications fournies par le personnel de santé. Selon l’étude menée au Bénin, 12,76 % des mères ignorent qu'il est nécessaire de compléter le calendrier vaccinal [5]. L'insuffisance d'explications, le manque d'amabilité des soignants et le temps d'attente excessif pourraient être corrigés par la sensibilisation du personnel de santé et sa formation à la relation avec les patients. Ceci devrait être un objectif prioritaire de tout programme d'amélioration de la couverture vaccinale pédiatrique au Niger et probablement dans toute l'Afrique subsaharienne.

Alors que 96,9 % de la population considère que c'est une bonne chose de faire vacciner ses enfants, les réponses positives sont de 88,4 % concernant le vaccin oral contre la poliomyélite. Ce résultat inférieur confirme que les journées nationales de vaccination contre la poliomyélite ont suscité une opposition spécifique contre ce vaccin [14].

Une proportion limitée de la population croit les rumeurs selon lesquelles des vaccins pédiatriques stériliseraient les filles (6,9 % pour les vaccins administrés dans les centres de santé et 7,3 % pour le vaccin oral des Journées nationales de vaccination contre la poliomyélite). Sans être élevés, ces niveaux sont cependant nettement supérieurs aux 2,2 % de réponses « Non » à la question « Est-ce une bonne chose de faire vacciner un jeune enfant? », ce qui semble paradoxal puisqu'un vaccin stérilisant ne saurait être considéré comme une bonne chose. Des études de psychologie ont montré que mentionner une rumeur la rappelle à des personnes qui l'avaient oubliée et lui confère de la crédibilité par la simple répétition de son existence [1, 3]. La formulation de la question susciterait ainsi des réponses en faveur de la rumeur, expliquant ces divergences.

Nos résultats apportent aussi un éclairage sur la gestion par la population des informations qu'elle reçoit sur les vaccins. Alors que les deux premières sources d'information sont l'entourage et les agents de santé, à égalité avec environ 61 %, la confiance dans l'entourage est la plus faible des six sources d'informations proposées (24,8 % de « Très confiants » et 40,9 % de « Méfiants »), alors que celle dans les agents de santé est de très loin la plus élevée (81,7 % de « Très confiants »). La population exerce un esprit critique sur les informations auxquelles elle est exposée,comme le montre également le décalage souligné précédemment entre le fait d'entendre et de croire une rumeur.

Enfin, notre étude fournit des explications sur le faible niveau de couverture vaccinale contre la Covid-19 au Niger et potentiellement en Afrique subsaharienne. Paradoxalement, 73,3 % de la population pensent que les vaccins contre la Covid-19 sont une bonne chose alors que moins d'un quart est vaccinée. Le plus remarquable est que 83,4 % des personnes qui ont entendu des messages de promotion de cette vaccination approuvent ces messages. La faible demande de vaccins contre la Covid-19 ne semble donc pas due à une mauvaise perception de ces vaccins.

La croyance dans les rumeurs négatives sur les vaccins contre la Covid-19 est limitée. La plus admise (26,1 %) est que les pays riches envoient en Afrique des vaccins dont ils ne veulent pas, rumeur qui peut trouver des fondements dans le don par des pays du Nord de vaccins AstraZeneca qu'ils n'utilisaient plus après l'arrivée des vaccins à ARN. Les pourcentages de la population croyant aux rumeurs qui attribuent des effets négatifs aux vaccins sont peu élevés : 9,6 % pensent que les vaccins provoquent des maladies, 7,9 % qu'ils stérilisent et 7,5 % qu'ils injectent une puce électronique. Comme nous l'avons vu précédemment ces réponses sont susceptibles d’être biaisées par le fait qu'en mentionnant ces rumeurs, les questions incitent à les approuver et leur prévalence réelle est probablement inférieure. Nos résultats montrent clairement que le manque d'adhésion à la vaccination contre la Covid-19 est dû à l'addition d'une série d'idées erronées sur l’épidémie. Plus de la moitié de la population pense que celle-ci ne touche pas le Niger et n'a donc aucune raison de se faire vacciner. Quant à la fraction qui sait que la maladie est présente, elle ignore presque totalement la transmission asymptomatique du virus et sa perception du risque est très faible : 87,6 % pensent que le coronavirus ne se trouve que chez des malades et 12,8 % seulement disent connaître quelqu'un qui a eu la maladie, proportion qui tombe à 5,5 % dans la famille.

Des travaux publiés à partir de 2022 ont montré une prévalence de la Covid-19 en Afrique subsaharienne beaucoup plus élevée que ce qu'on pensait précédemment. Au Niger, alors que fin 2021, 275 décès dus à la Covid-19 étaient rapportés dans le pays depuis le début de la pandémie, le nombre estimé par une étude était 14 222 et l'excès de mortalité, qui inclut les décès indirects dus à une diminution de l'offre de soins, était de 9 622 selon l'OMS et de 18 100 selon une autre étude [4, 10, 11, 15]. Les informations fournies à la population sur la morbidité et la létalité de la Covid-19 sousestiment ainsi très largement leur fardeau réel. La population nigérienne ignore la réalité de la Covid-19 dans le pays et pense très largement ne jamais avoir croisé de personne infectée, ni de malade. Elle a vu sur les réseaux sociaux et sur des chaînes de télévision étrangères des reportages sur la morbidité et la létalité de la maladie dans le reste du monde, alors que de tels reportages sur le pays étaient quasiment inexistants. Dans ces conditions, la vaccination contre la Covid-19 peut paraître aussi inutile à la majorité des Nigériens que celle contre la fièvre jaune à un Européen qui ne quitte pas l'hémisphère nord.

## Conclusion

Cette étude était destinée à fournir des données sur lesquelles appuyer une stratégie de communication pour le renforcement de la vaccination. Ses résultats, parfois inattendus, peuvent probablement s'appliquer à l'ensemble de l'Afrique de l'Ouest.

Pour les vaccins du PEV, auxquels la quasitotalité de la population adhère, la communication doit s'attacher à expliquer les causes des effets indésirables des vaccins et leur gestion, et sensibiliser à l'importance du respect du calendrier vaccinal. Parallèlement, un effort de formation doit être déployé auprès du personnel de santé pour améliorer l'accueil des mères qui viennent faire vacciner leurs enfants, afin qu'il fasse preuve d'amabilité et réduise les temps d'attente.

Le faible taux de vaccination contre la Covid-19 étant principalement dû à des idées erronées sur l’épidémie et non sur les vaccins, l'enjeu de la communication sur cette vaccination est de faire prendre conscience de la réalité de la maladie dans le pays et de sensibiliser sur les groupes à risque de formes graves.

## Financement

Ce travail a été financé par l'Agence française de développement, dans le cadre du programme AFROVAX mené par la Croix-Rouge française au Niger, en Guinée et aux Comores.

## Remerciements

Nous remercions les enquêteurs, qui ont mené les entretiens individuels et les groupes focus, et rempli les questionnaires.

## Contribution des auteurs

Bernard SEYTRE : conception, analyse des résultats, rédaction

Sanoussi CHAIBOU : conduite des enquêtes, analyse des résultats, rédaction Emmanuel CHABOT : analyse des résultats, rédaction

Bernard SIMON : conception, analyse des résultats, rédaction

## Liens d'intérêt

Les auteurs ne déclarent aucun lien d'intérêt.
